# Peroxiporin Expression Is an Important Factor for Cancer Cell Susceptibility to Therapeutic H_2_O_2_: Implications for Pharmacological Ascorbate Therapy

**DOI:** 10.1371/journal.pone.0170442

**Published:** 2017-01-20

**Authors:** Dieanira Erudaitius, Andrew Huang, Sarah Kazmi, Garry R. Buettner, Victor G. J. Rodgers

**Affiliations:** 1 Department of Bioengineering, University of California, Riverside, Riverside, California, United States of America; 2 Department of Neuroscience, University of California, Riverside, Riverside, California, United States of America; 3 Free Radical & Radiation Biology, Department of Radiation Oncology, University of Iowa College of Medicine, Iowa City, IA, United States of America; University of South Alabama Mitchell Cancer Institute, UNITED STATES

## Abstract

Cancer cell toxicity to therapeutic H_2_O_2_ varies widely depending on cell type. Interestingly, it has been observed that different cancer cell types have varying peroxiporin expression. We hypothesize that variation in peroxiporin expression can alter cell susceptibility to therapeutic H_2_O_2_ concentrations. Here, we silence peroxiporin aquaporin-3 (AQP3) on the pancreatic cancer cell line MIA PaCa-2 and compare clonogenic survival response to the wild-type. The results showed a significantly higher surviving fraction in the clonogenic response for siAQP3 MIA PaCa-2 cells at therapeutic H_2_O_2_ doses (*P* < 0.05). These results suggest that peroxiporin expression is significant in modulating the susceptibility of cancer cells to ascorbate therapy.

## Introduction

Recent preclinical studies and a Phase I clinical trial [1–4] have demonstrated promise in the use of the pro-drug pharmacological ascorbate (P-AscH^-^) as an adjuvant in the treatment of pancreatic ductal adenocarcinoma. Intravenous infusions of P-AscH^-^ (plasma concentrations of ≈20 mM) decreased tumor volume and suggested increased survival of patients with stage 4 pancreatic cancer [3]. P-AscH^-^ has promise for improving outcomes for pancreatic cancer patients; however, its broad application for other types of cancer has yet to be realized. The impotence in moving forward with P-AscH^-^ therapy for patients with other types of cancer is due, in part, to observations in a recent *in vitro* study by Chen *et al*. (2008) [5]. There, they reported that while normal cells remain relatively unaffected to P-AscH^-^, cancer cell lines exhibit a wide range of responses as seen by rates of clonogenic survival. Therefore, it is of great interest to understand why certain cancer cells are more responsive to P-AscH^-^ and thereby guide the use of P-AscH^-^ as an adjuvant to cancer therapy.

### Extracellular H_2_O_2_ is Primary Factor in Ascorbate Therapy Efficacy

The mechanism behind ascorbate mediated cell death is being studied extensively [1, 5–9]. The work developed from understanding the unique nature of ascorbate and its ability to serve as an anti-oxidant (at low physiological concentrations) compared to its pro-oxidant behavior (at pharmacological concentrations) [5, 10]. It is now clear that at higher concentrations (achievable through intravenous administrations), ascorbate undergoes autoxidation and readily forms H_2_O_2_. Furthermore, the presence of catalytic metals (i.e. iron) serves to accelerate this process [11]. In addition to the biochemical nature of ascorbate, a tremendous amount of work has been directed into discovering the variation in enzymatic activity of ascorbate-susceptible cells [10, 12]. In order to unveil the mechanism of ascorbate-mediated cell death, a significant amount of research has worked on understanding the type of cell death induced (apoptosis, autophagy, etc.) [10, 13] and the intracellular damage (metabolic, nuclear, etc.) [12] that occurs. It was further confirmed that cytotoxicity was a result of extracellular and not intracellular ascorbate [14, 15]. Furthermore, numerous *in vivo* and *in vitro* studies have displayed a range of susceptibility to P-AscH^-^ across different types of cancer [1, 5, 13, 15–24], and intracellular H_2_O_2_, being the byproduct of P-AscH^-^ oxidation, has been identified as the primary factor for cellular cytotoxicity.

Thus, ascorbate is categorized as a pro-drug due to its ability to generate high concentrations of extracellular hydrogen peroxide (H_2_O_2_) that permeates into the intracellular space [4, 10, 14, 15]. It has been demonstrated that the effects of P-AscH^-^ are reversible with the introduction of specific H_2_O_2_ scavenging enzymes [25], further supporting the argument that extracellular H_2_O_2_ is the primary factor in cytotoxicity via P-AscH^-^. More specifically, the effect of P-AscH^-^ on pancreatic cancer cells was found to be mitigated when co-cultured with catalase (the primary scavenging enzyme in the presence of high H_2_O_2_ concentrations) [5, 12]. Doskey et al. (2016) [12] demonstrate that H_2_O_2_ is involved in the mechanism of P-AscH^-^ toxicity to cancer cells and that the removal of H_2_O_2_ via catalase is an important factor.

The extracellular H_2_O_2_ generated by ascorbate ultimately permeates across the plasma membrane. This, in turn, increases the intracellular H_2_O_2_ [25] to substantially higher levels than physiological concentrations. Extracellular P-AscH^-^ has also been shown to induce DNA damage (mitochondrial and nuclear) in addition to ATP depletion via H_2_O_2_ [1, 2, 13, 15, 22–24, 26–27]. Again, introducing extracellular catalase to the P-AscH^-^ culture prevented ATP depletion which supports the hypothesis that ascorbate-mediated ATP depletion is via the extracellular H_2_O_2_ produced that permeates the cell. At these elevated concentrations, in addition to the DNA damage and ATP level effects that occur, it has also been suggested that intracellular H_2_O_2_ is activated in the presence of catalytic transition metals generating significant hydroxyl radical (HO^•^) [28]. Ultimately, this high flux of HO^•^ substantially increases DNA damage, which is believed to be the primary factor in inhibiting cellular reproduction. Doskey et al. (2016) [12] show that the ED_50_ results for clonogenic exposure to P-AscH^-^ is directly coupled to the rate of H_2_O_2_ uptake per cell. This finding confirms that H_2_O_2_ is a primary factor in DNA damage as well as in compromising ATP levels during P-AscH^-^.

### Cellular Properties that Effect Therapeutic H_2_O_2_ Intracellular Concentrations

#### Catalase activity

Among the various scavenging enzymes that control the intracellular H_2_O_2_ concentration at physiological conditions [10], catalase appears to be the primary enzyme contributing to the removal of the H_2_O_2_ generated by P-AscH^-^ [10, 12, 29–31]. Interestingly, catalase exhibits higher activity in normal cells where its expression can range on the order of 10- to 100-fold greater than in some tumor cells [32]. This difference in catalase activity amongst cells can greatly affect the rate of intracellular removal of H_2_O_2_ generated by P-AscH^-^. It is believed that the high catalase activity of normal cells reduces the intracellular H_2_O_2_ concentrations to levels that are non-toxic. Conversely, tumor cells with relatively low catalase activity are expected to be more susceptible to ascorbate-mediated cell-death.

#### Plasma membrane permeability

The variability in the plasma membrane permeability to H_2_O_2_ may be another factor that contributes to the fate of cells upon exposure to P-AscH^-^. Like catalase activity, plasma membrane permeability to H_2_O_2_ also exhibits significant variability across cell lines *via* the wide range of expression levels of peroxiporins. Peroxiporins are aquaporins (AQPs) that facilitate the flux of H_2_O_2_ across the plasma membrane [33, 34]. The AQP isoforms currently identified that allow passive transport of H_2_O_2_ are AQP1, AQP3, and AQP8 [33, 35]. AQPs are heavily expressed in many types of tumors [36], especially those considered aggressive [37]. Thus, it is hypothesized that increased plasma membrane permeability to extracellular H_2_O_2_ (*i*.*e*., *via* enhanced expression of peroxiporins) can further increase the efficacy of P-AscH^-^ therapy.

In this work, we investigate the significance of plasma membrane H_2_O_2_ permeability to *in vitro* cell susceptibility to therapeutic extracellular H_2_O_2_ concentrations. In particular, the clonogenic surviving fraction response for the pancreatic cancer cell line MIA PaCa-2 with modified peroxiporin expression is evaluated. Initially the expression of AQP1, AQP3, and AQP8 of MIA PaCa-2 are qualitatively screened against the normal pancreatic tissue cell line H6c7 using an immunocytochemistry assay. Recognizing that AQP3 is substantially overexpressed in MIA PaCa-2, the study focuses on silencing AQP3 (siAQP3 MIA PaCa-2). Next the relative expression levels of AQP3 for both siAQP3 MIA PaCa-2 and unmodified MIA PaCa-2 using flow cytometry is verified. In addition, the rate of H_2_O_2_ uptake and cell susceptibility between the two cell lines are compared. Finally, the clonogenic surviving fraction for exposure to therapeutic H_2_O_2_ concentrations is evaluated for siAQP3 MIA PaCa-2 and unmodified MIA PaCa-2. The results of this study show that AQP3 expression is significant in the clonogenic surviving fraction response for MIA PaCa-2 for *in vitro* therapeutic exposure to H_2_O_2_. These results emphasize the importance of considering plasma membrane permeability to H_2_O_2_ when elucidating cellular properties that can impact the response of cells to exposure to extracellular H_2_O_2_ and the success of P-AscH^-^ as an adjuvant to cancer therapies.

## Materials and Methods

### Antibodies

The following antibodies were used in this study for immunocytochemistry and flow cytometry: rabbit anti-AQP1 antibody (SAB5200109; Sigma Aldrich, St. Louis, MO, USA), rabbit anti-AQP3 antibody (SAB5200111; Sigma Aldrich, St. Louis, MO, USA), mouse anti-AQP8 antibody (SAB1403559; Sigma Aldrich, St. Louis, MO), goat anti-rabbit IgG (A11008; Life Technologies, Carlsbad, CA, USA), goat anti-mouse IgG (A11005; Life Technologies, Carlsbad, CA, USA).

### Cells and Reagents

Pancreatic H6c7 cells (HPV16-E6E7) [38] were established by transduction of HPV16-E6E7 genes into a primary culture of normal pancreatic duct epithelial cells and cultured in keratinocyte SFM (KSFM, Invitrogen, Carlsbad, CA) with supplements: human recombinant epidermal growth factor and bovine pituitary extract (Life Technologies, Carlsbad, CA, USA), in addition to 1% antibiotics. Pancreatic adenocarcinoma MIA PaCa-2 cells (American Type Culture Collection Manassas, VA) were cultured in Dulbecco’s Modified Eagle’s Medium (DMEM, Life Technologies, Carlsbad, CA, USA) with 10% fetal bovine serum (FBS, Life Technologies, Carlsbad, CA, USA) and 1% antibiotic. All cells were maintained at incubation of 37°C and supplied with 5% CO_2_ and 1% penicillin streptomycin (Life Technologies, Carlsbad, CA, USA).

### Immunocytochemistry Staining

Cells were seeded on glass cover slips (ThermoFisher Scientific, Lafayette, CO, USA) 48 h before fixing with paraformaldehyde (4% PFA) for 15 min. PFA was removed by three 5-min 1x PBS washes. Normal goat serum (5% NGS) diluted in 1x PBS was added to cells for 1 h at room temperature (RT) on a shaker to block non-specific binding. Primary antibodies diluted 1:200 in 0.3% Triton X 100 (in PBS) were added to cells and left to gently shake for 12 h in 4°C. Primary antibodies were removed *via* three 5-min 1x PBS washes. Secondary antibodies diluted 1:100 in NGS were added to cells and placed on shaker for 2 h RT. Secondary antibodies were removed by three 5-min 1x PBS washes and glass coverslips containing stained cells were mounted on microscope slides (ThermoFisher Scientific, Lafayette, CO, USA). NucBlue Live Cell Stain ReadyProbes reagent (R37605; Life Technologies, Carlsbad, CA, USA) was added to stain the nucleus of cells. Images were taken with the Lecia SP5 confocal microscope (Lecia, Solms, Germany) and analyzed using ImageJ (NIH). AQP 1, 3 or 8 was determined by measuring target fluorescence intensity (from 11 images) per cell area for H6c7 and MIA PaCa-2 cells. Statistical significance between protein expression (AQP 1, 3 or 8) and each cell type was determined through ANOVA (Single Factor). P-values less than 0.05 were accepted as indicating a statistical significant difference. Error bars represent standard error (SE). Data were analyzed and plotted using Excel-2007 (Microsoft; Redmond, WA), and SigmaPlot (Systat Software Inc; San Jose, CA, USA) software.

### Silencing AQP3 on MIA PaCa-2 Cells

Silencing was accomplished through reverse transfection using double stranded siRNA, siRNA AQP3 (s1523; Invitrogen, Carlsbad, CA). The protocol provided by Invitrogen was adjusted appropriately. A total of 6 pmol siRNA AQP3 (20 μL or 500 μL) were diluted in Opti-MEM I Reduced Serum Medium (31985–062; Life Technologies, Carlsbad, CA, USA) then added and evenly spread in wells. Following with the addition of Lipofectamine RNAiMAX (133778–150; Life Technologies, Carlsbad, CA, USA) (0.3 μL or 5 μL) and thoroughly mixed to each, 96- or 6-well plates, containing the diluted siRNA molecules. The siRNA and Lipofectamine were allowed to interact 10–20 min at RT to allow siRNA-lipid complex formation. Cells were diluted in appropriate complete growth medium and cell density reached 30–50% confluency 24 h after plating. Plates were gently mixed. Plates were incubated for 48 h at 37°C with 5% CO_2_ supplied. The transfection efficiency was obtained for MIA PaCa-2 using siRNA Cy-3 GAPDH (Life Technologies, Carlsbad, CA, USA) in place of siAQP3 strands and serves as a positive control for the method of silencing. Successfully transfected cells were visualized using ArcturusXT LCM System (ThermoFisher Scientific, Lafayette, CO, USA) and counted using disposable hemocytometers (INCYTO, Covington, GA, USA). Scrambled siRNA AQP3 (4390843; Life Technologies, Carlsbad, CA, USA) sequences were used for the negative control of each experimental set up to ensure that the silencing procedure was not affecting results. The scrambled siRNA sequences were delivered to the cells in an identical manner as the siRNA AQP3 with the same concentrations of each component and identical cell plating number.

### Relative AQP3 Expression

Flow cytometric analysis was performed to obtain quantification in fluorescent signal reduction for AQP3 between MIA PaCa-2 unmodified and siAQP3 MIA PaCa-2 cells. Cells were harvested using accutase (A6964; Sigma Aldrich, St. Louis, MO, USA) and quenched using cell culture medium consisting of DMEM (Life Technologies, Carlsbad, CA, USA) with 10% FBS (Life Technologies, Carlsbad, CA, USA) and 1% antibiotic. Cells were centrifuged (1000 rpm) (Marathon 8K centrifuge; Beckman Coulter, Indianapolis, IN, USA) for 5 min at RT. Following centrifugation, cells were re-suspended in ice-cold FACS Buffer (5 mL) containing D-PBS (14190250; ThermoFisher Scientific, Lafayette, CO, USA), BSA (0.5% w/v final) (A7906; Sigma Aldrich, St. Louis, MO, USA), and 2 mM EDTA (15575020; ThermoFisher Scientific, Lafayette, CO, USA) and centrifuged (1000 rpm) (Eppendorf centrifuge 5415R, Hauppauge, NY, USA) for 5 min at RT. Subsequently, cells were re-suspended in ice-cold FACS Buffer (100 μL) and further labelled with rabbit anti-AQP3 antibody (1:25 dilution), gently mixed, and incubated for 20 min at 4°C. Cells were then centrifuged (1000 rpm) (Eppendorf centrifuge 5415R, Hauppauge, NY, USA) twice for 4 min at RT with a 1 mL ice-cold FACS buffer wash in between. A fluorescence labeled anti-rabbit IgG (DI-1488; Vector Laboratories) was added (1:100 dilution), mixed gently, and incubated for 15 min at 4°C. Cells were then centrifuged (1000 rpm) (Eppendorf centrifuge 5415R, Hauppauge, NY, USA) twice for 4 min at RT with a wash using FACS buffer in between the spins. Subsequently, cells were fixed with 1% PFA and detected by a Cell Lab Quanta SC flow cytometer (Beckman Coulter, Brea, CA, USA). Data was analyzed and plotted with FlowJo (Treestar, Inc., Ashland, OR, USA).

### Rate of H_2_O_2_ Uptake Per Cell

The rate of H_2_O_2_ uptake for unmodified MIA PaCa-2 and siAQP3 MIA PaCa-2 cell lines were measured, in the same manner as described previously by Wagner *et al*. [39]. This assay provides an exogenous H_2_O_2_ removal rate, on a per cell basis. The assay measures the change in extracellular H_2_O_2_ over time, which decays exponentially representing a pseudo-first order behavior of the intracellular catalase reaction. The technique is a highly sensitive fluorescent method capable of detecting low concentrations of H_2_O_2_, below 0.5 μM. Briefly, cells were seeded in 96-well culture (Corning, Union City, CA, USA) treated dishes and incubated 48 h prior to the assay at 37°C, 5% CO_2_; 90% confluency was reached. An extracellular bolus of 20 μM H_2_O_2_ (Sigma, St. Louis, MO, USA) was introduced in 5 min intervals to defined wells containing cells. A quenching solution comprised of 20 mL 1x HBSS (ThermoFisher Scientific, Lafayette, CO, USA), 20 μL 1M 4(-2-hydroxyethyl)-1-piperazineethansulfonic acid (HEPES) (pH 7.2–7.5) (ThermoFisher Scientific, Lafayette, CO, USA), 10 mg NaHCO_3_ (3mM) (ThermoFisher Scientific, Lafayette, CO, USA), 5 mg 4-hydroxyphenylacetic acid (*p*HPA) (Sigma, St. Louis, MO, USA), and 2 mg HRP (horse radish peroxidase Type 1) (Sigma, St. Louis, MO, USA) was used to terminate the assay. The quenching solution prevents any remaining H_2_O_2_ from entering the cell as H_2_O_2_ instead activates HRP which in turn oxidizes *p*HPA resulting in the fluorescent *p*HPA dimer. The fluorescent signal is representative of the H_2_O_2_ concentration in each well and is further detected via the Tecan F200 (Tecan US, Morrisville, NC) plate reader with an excitation at 340 nm (bandwidth 20 nm) and monitoring an emission at 430 nm (bandwidth 20 nm) from above the wells. Wells containing cells were trypsinized and the number of cells were determined using a Moxi Z Mini Automated Cell Counter (ORFLO Technologies, Ketchum, ID, USA). The capacity of the cells to remove extracellular H_2_O_2_ (*k*_*cell*_) is calculated from the number of cells, concentration of H_2_O_2_ remaining, total volume of media, and the observed rate of extracellular H_2_O_2_ removal (*k*_*obs*_). Statistical significance between *k*_*cell*_ was determined through ANOVA (Single Factor) and the presented errors were propagated. Since *k*_*cell*_ has two associated errors, *k*_*obs*_ (obtained through linear regression) and the number of cells, the errors in *k*_*cell*_ were propagated. Cells were counted at the end of the experiment. P-values less than 0.05 were accepted as indicating a statistical significant difference. Data were analyzed and plotted using Excel-2007 (Microsoft; Redmond, WA), and SigmaPlot *(*Systat Software Inc; San Jose, CA, USA) software.

### Clonogenic Assessment

Cells (2 × 10^5^) were seeded in 6-well culture (Corning, Union City, CA, USA) treated dishes and exposed to appropriate H_2_O_2_ doses 48 h later. H_2_O_2_ exposures of (0 nmol cell^-1^–0.30 nmol cell^-1^; representative of 0, 50, 60, 70, 80 and 90 μM) [40] were diluted in the appropriate culture media and cells were exposed for 1 h at 37°C. After exposure, the diluted media was removed, cells were trypsinized and counted with a Moxi Z Mini Automated Cell Counter (ORFLO Technologies, Ketchum, ID, USA) and re-plated at 300 cells mL^-1^ in triplicates with appropriate media in 6-well culture (Corning, Union City, CA, USA) treated dishes. Plates were incubated for two weeks at 37°C, 5% CO_2_ and colonies formed between 10 to 14 d at 37°C. Following a two-week incubation period, the colonies were fixed with 70% ethanol and stained with Coomasie Brilliant Blue R-250 (1610436; BioRad, Hercules, CA). Colonies with more than 50 cells were counted using a Counter-Pen (3133; Traceable Products, Webster, TX). The plating efficiency (PE) and surviving fraction (SF) were determined; PE = (colonies counted/cells plated) x 100 and SF = (PE of treated sample/PE of control) x 100 [41, 42]. Statistical significance between each H_2_O_2_ exposure dose and cell types or cell modification was determined through ANOVA (Single Factor). P-values less than 0.05 were accepted as indicating a statistical significant difference. Error bars displayed represent the standard error (SE). Data were analyzed and plotted using Excel-2007 (Microsoft; Redmond, WA), and SigmaPlot (Systat Software Inc; San Jose, CA, USA) software. Plots of H_2_O_2_ exposure doses are represented in the nmol cell^-1^ instead of concentrations because it serves as a more informative dosing metric for cell culture, as often times variations seen in experimental results arise as these systems are cell density dependent [40].

## Results

### Immunocytochemistry Staining for Peroxiporins

**Fig 1:**
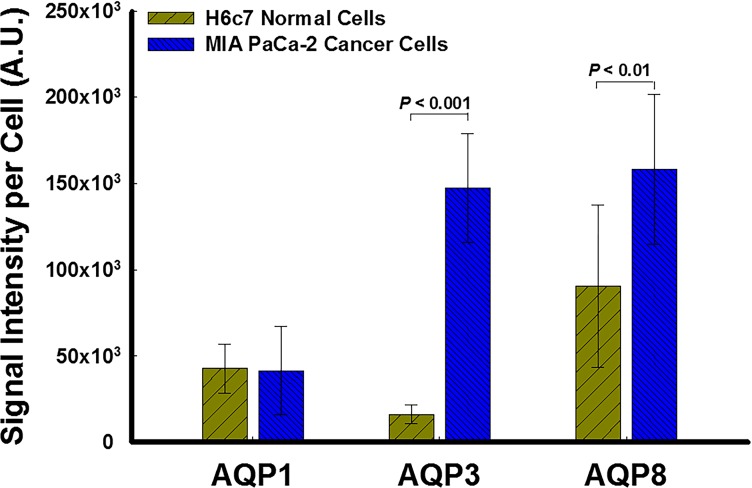
Pancreatic cancer cells exhibit elevated peroxiporin (AQP3 and AQP8) expression compared to normal cells. The green fluorescence signal intensity from immunocytochemistry staining for the presence of peroxiporins images were analyzed per cell using ImageJ (NIH). Elevated signal intensities indicate a greater presence of a protein and therefore elevated expression. Elevated expression of peroxiporin AQP1 is not apparent between H6c7 and MIA PaCa-2 cells. However, both peroxiporin AQP3 and AQP8 show a significant difference between MIA PaCa-2 and H6c7 cells (*P* < 0.001 and *P* < 0.01, respectively, *n* = 3 in both cases). P-values are obtained through ANOVA. Error bars represent standard error (SE). A.U. = arbitrary units.

We conducted immunocytochemistry staining to verify the presence of peroxiporins AQP1, AQP3, and AQP8 for MIA PaCa-2 and H6c7 cells. In addition to verifying the presence of these peroxiporins, the signal intensities evaluated from immunocytochemistry also allowed for a qualitative measurement for the relative expression levels for each peroxiporin on both cell types. Elevated signal intensities indicate greater presence of these proteins and therefore elevated expression. Images of the immunocytochemistry staining for AQP1, AQP3, and AQP8 in H6c7 and MIA PaCa-2 cell, allow qualitative assessment for AQP expression of each cell type. Quantification shows the variation in expression of each of the AQPs between the two cell-types providing insight as to which peroxiporin is more highly expressed by MIA PaCa-2 cancer cells, Fig 1. Differences in expression of peroxiporin AQP1, is not apparent between H6c7 and MIA cells. Although AQP8 has a higher expression in MIA PaCa-2 compared to H6c7 cells, it is clear that AQP3 is significantly more elevated in MIA PaCa-2 cells compared to H6c7 cells. This study therefore focuses on the significance of AQP3.

### Silencing Reduces AQP3 on the Plasma Membrane

**Fig 2:**
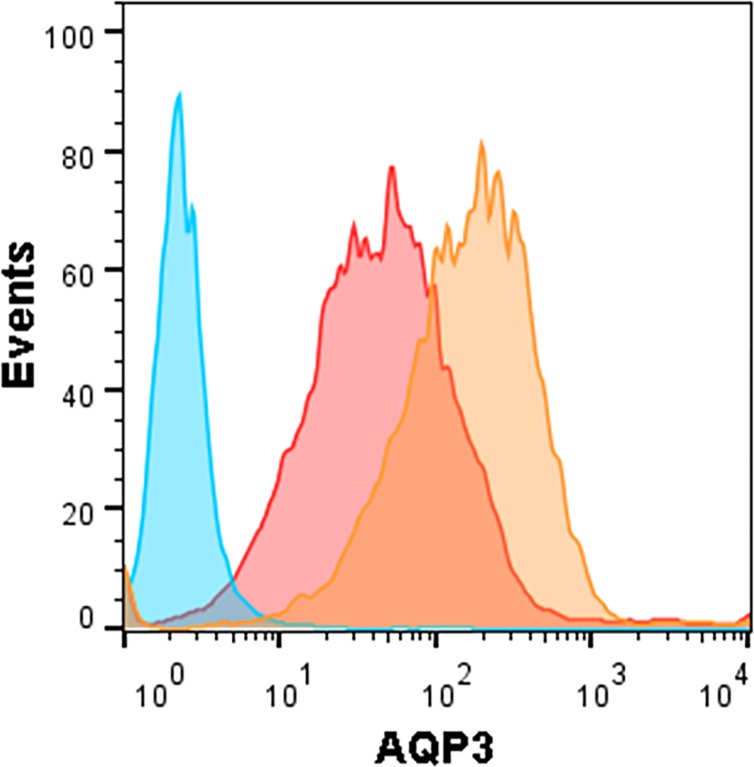
Silencing AQP3 with siRNA reduces AQP expression by a factor of 10 for MIA PaCa-2 cells. Verification for silencing of peroxiporin AQP3 on the plasma membrane of cancer cells is confirmed by the observed shift in the AQP3 apparent signal. An AQP3 specific signal is shown to exhibit a positive frequency of 91.7 (orange curve) for the unmodified MIA PaCa-2 cancer cells sampled (8,036 cells). After silencing AQP3 for MIA PaCa-2 cancer cells (siAQP3 MIA PaCa-2), the signal shifts to display a positive signal frequency of 59.8 (red curve) for sampled cells (8,067 cells). The peak of the unmodified MIA PaCa-2 cells (orange) displays an average positive signal around 200 whereas the siAQP3 MIA PaCa-2 cells (red) displays an average around 20. This shift in AQP3 signal demonstrates a decrease by a factor of 10 in AQP expression for the silenced MIA PaCa-2 cells when compared to unmodified MIA PaCa-2. The blue curve is the negative IgG control. Data were generated by immunofluorescence tagging, detection through flow cytometry, and analyzed through FlowJo (Treestar, Inc., Ashland, OR, USA).

To examine the role of AQP3 in modulating the rate of uptake of extracellular H_2_O_2_ by MIA PaCa-2 cells we used siAQP3 as a tool to modulate AQP3 expression. Flow cytometric analysis was used to verify the silencing of peroxiporin AQP3 on the plasma membrane. We were able to confirm a factor of 10 relative decrease in peroxiporin AQP3 expression for the silenced cells by obtaining AQP3 specific signals for wild-type unmodified MIA PaCa-2 *vs*. silenced AQP3. Fig 2 shows a positive AQP3 signal frequency of 91.7 (orange curve) for the unmodified MIA PaCa-2 cancer cells sampled (8,036 cells). After silencing AQP3 for MIA PaCa-2, the signal shifts to display a positive signal frequency of 59.8 (red curve) for the silenced MIA PaCa-2 sampled cells (siAQP3 MIA PaCa-2, 8,067 cells). The shift in fluorescence between MIA PaCa-2 (orange) and siAQP3 MIA PaCa-2 cells (red) demonstrates a decrease in AQP3 expression for siAQP3 MIA PaCa-2 cells by a factor of 10, when comparing the average displayed by the peaks of each curve.

### Rate of Uptake of Extracellular H_2_O_2_

**Fig 3:**
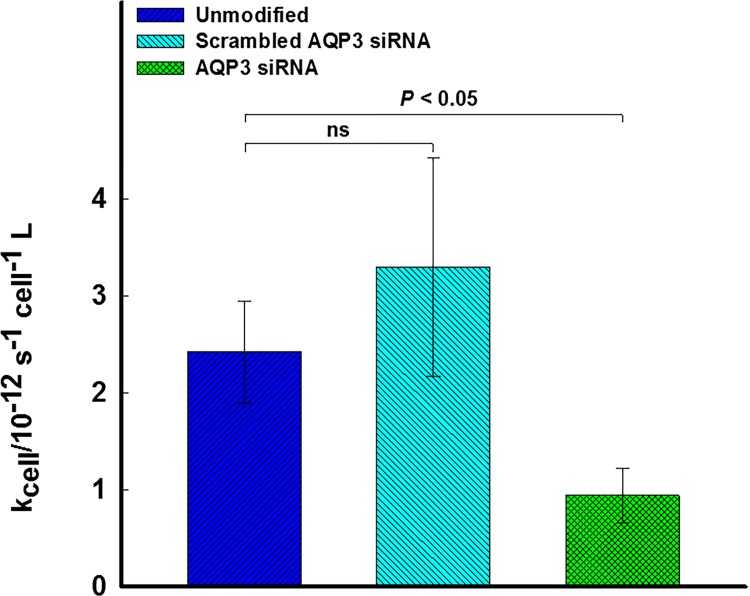
Silencing AQP3 on MIA PaCa-2 cancer cells decreases the rate of H_2_O_2_ uptake per cell. The rate of H_2_O_2_ uptake by per cell is displayed for MIA PaCa-2 (blue) and siAQP3 MIA PaCa-2 (green). There is a significant decrease in rate between MIA PaCa-2 and siAQP3 MIA PaCa-2 cells (*P* = 0.002, *n* = 4). The rate of H_2_O_2_ uptake for the negative control (scrambled MIA PaCa-2, cyan) is not significantly different from MIA PaCa-2 (*P* = 0.41, *n* = 4) confirming that the silencing method is not affecting the results. P-values are displayed for cases that are significantly different and are determined through ANOVA analysis. Error bars displayed represent the propagated error.

The rate of exogenous H_2_O_2_ uptake was determined for MIA PaCa-2 and siAQP3 MIA PaCa-2 cells using a kinetic assay described previously [39]. The rate constant for the uptake of extracellular H_2_O_2_ per cell is significantly decreased for siAQP3 MIA PaCa-2 as compared to MIA PaCa-2 cells (*P* = 0.002, *n* = 4), Fig 3. This confirms that AQP3 is an important factor in controlling the flux of H_2_O_2_ through the plasma membrane. Scrambled siRNA for AQP3 (negative control) displayed no significant difference for the rate of H_2_O_2_ uptake when compared to MIA PaCa-2 unmodified cells (*P* = 0.41, *n* = 4).

### Clonogenic Survival is Increased when AQP3 is Silenced

**Fig 4:**
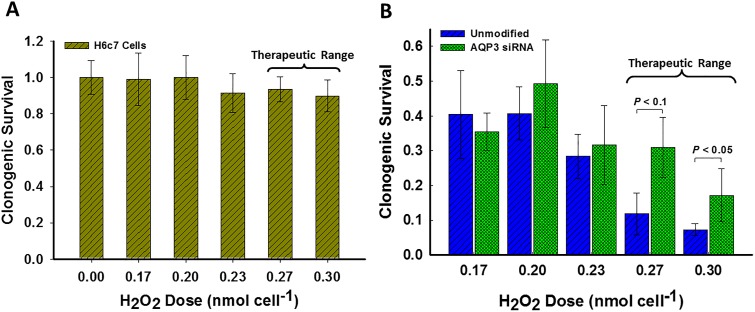
Silencing AQP3 increases the surviving fraction of pancreatic cancer cells at therapeutic H_2_O_2_ concentrations. A) Surviving fraction, relative to 0 μM H_2_O_2,_ of H6c7 cells are not significantly affected for dosing shown. B) Surviving fraction, relative to 0 μM H_2_O_2_, is significantly increased for siAQP3 MIA PaCa-2 as compared to unmodified MIA PaCa-2 for therapeutic dosing of 0.27 nmol cell^-1^ (80 μM) and 0.30 nmol cell^-1^ (90 μM) H_2_O_2_ (*P* = 0.08 and 0.02, respectively and *n* = 3 for both). Surviving fraction of H6c7 cells are not significantly affected for dosing shown. These results imply that plasma membrane permeability to H_2_O_2_ *via* AQP3 is an important factor in the surviving fraction outcomes for MIA PaCa-2. Statistical significance was determined through ANOVA. Error bars displayed represent the standard error (SE).

Assays designed to determine the clonogenic survival of cells upon exposure to a bolus of H_2_O_2_ up to 0.30 nmol cell^-1^ (corresponding to a concentration of 90 μM) reveal the dose-response for the three cell lines, Fig 4A and 4B. H6c7 cells (Fig 4A) were unaffected by exposure to bolus addition of extracellular H_2_O_2_. MIA PaCa-2 and siAQP3 MIA PaCa-2 cells both demonstrated significant decrease in their surviving fraction when exposed to increased concentrations of H_2_O_2_ compared to their controls, Fig 4B. However, siAQP3 MIA PaCa-2 cells showed an increase in surviving fraction compared to MIA PaCa-2 cells upon exposure to therapeutic ranges of H_2_O_2_ at 0.27 nmol cell^-1^ (80 μM) (*P* = 0.08, *n* = 3) and 0.30 nmol cell^-1^ (90 μM) (*P* = 0.02, *n* = 3). These results indicate that the AQP3, which facilitates the permeability of H_2_O_2_ across the plasma membrane, is an important determinant of the toxicity of H_2_O_2_; AQP3 expression appears to be a significant factor in the outcome of ascorbate therapy.

## Discussion

While pancreatic cancer cells exhibit significantly reduced proliferation in the presence of extracellular P-AscH^-^ [1], and normal cells remain unaffected, other cancer cells exhibit a wide variation in susceptibility. Previously, a focus for the underlying differences in susceptibility to P-AscH^-^ has been on the varying catalase activity across cell types [1, 10]. Catalase serves as an intracellular sink for the H_2_O_2_ generated by P-AscH^-^. Our results show that AQP3 acts as a conduit for the flux of H_2_O_2_ into the cell [10]. However, a more complete analysis is required for understanding overall flux contributions from variation in permeability to H_2_O_2_ as well as catalase activity.

The overall intracellular concentration of H_2_O_2_ in normal cells is likely to be substantially less than in pancreatic cancer tumor cells during therapy with P-AscH^-^. Thus, the removal rate of H_2_O_2_ is likely to be substantially higher for H6c7 cells as opposed to MIA PaCa-2 once it has crossed the plasma membrane. But, in addition, the results from Fig 1 imply that the permeability of H_2_O_2_, at least through the available peroxiporins, is also substantially reduced for H6c7 cells as compared to MIA PaCa-2. This further suggests that the expression of peroxiporins may be also linked to the susceptibility of cells to the H_2_O_2_ generated by P-AscH^-^. Thus, those cancer cells that are most susceptible may have an increased expression of peroxiporin in addition to a lower relative catalase activity compared to normal cells.

We show that silencing a peroxiporin, specifically AQP3, inhibits the passage of H_2_O_2_ into the cell. Additionally, and more importantly, the silencing of peroxiporin AQP3 on pancreatic cancer cells suggests that accumulation of lethal intracellular H_2_O_2_ concentrations is prevented; consequently, allowing for an increase in clonogenic response. Silencing peroxiporin AQP3 resulted in an increase in surviving fraction of siAQP3 MIA PaCa-2 cells in a clonogenic assay using pharmacological H_2_O_2_ concentrations of 0.30 nmol cell^-1^ (90 μM) in comparison to MIA PaCa-2 (*P* = 0.02). This implies that cell-susceptibility to ascorbate therapy is significantly coupled to the permeability of the cell’s plasma membrane to H_2_O_2_, and in particular, elevated expressions of peroxiporins.

Susceptibility to P-AscH^-^ is mirrored in clonogenic assays in response to therapeutic H_2_O_2_ *in vitro* [1]. Therapeutic H_2_O_2_ levels range between 0.27 nmol cell^-1^ to 0.30 nmol cell^-1^ (80 μM to 90 μM) and is representative of extracellular H_2_O_2_ produced upon delivery of P-AscH^-^. In a murine model when P-AscH^-^ is given intravenously, concentrations on the order of 20 μM of extracellular H_2_O_2_ can be achieved [2]. Thus, clonogenic assays are appropriate assessment in this work. The therapeutic range of H_2_O_2_ for the clonogenic studies was between 80 μM and 90 μM. This is consistent with 87 μM of extracellular H_2_O_2_ achieved following intravenous P-AscH^-^ infusions [15]. In that study the extracellular ascorbate reached 34 mM.

Overall, this work demonstrates that the permeability of the plasma membrane to H_2_O_2_ is an important factor when addressing the efficacy of P-AscH^-^ as an adjuvant to cancer therapy. Although extensive research would be required, modulating membrane peroxiporin expression may increase the efficacy of P-AscH^-^ as an adjuvant for other types of cancer. As a side note, some drugs, such as gemcitabine used for pancreatic cancer, are known to elevate peroxiporin, specifically AQP3, expression in cancer cells [3, 43]. This additional factor may be significant for expanding the use of P-AscH^-^ therapy for other forms of cancer.

### Implications for Ascorbate Therapy

Extracellularly, ascorbate generates H_2_O_2_ that ultimately permeates across the plasma membrane. This H_2_O_2_, if not adequately removed by the cell, results in intracellular H_2_O_2_ accumulation that prevents the cell from remaining viable. The work presented here, demonstrates that peroxiporin expression is potentially an additional and important factor in determining the success of pharmacological ascorbate therapy. It is suggested that cancer cells with elevated peroxiporins on the plasma membrane could provide increased routes of entry for H_2_O_2_ which could potentially contribute to intracellular H_2_O_2_ accumulation. Since many cancer tissues and cells have elevated expressions of AQPs, further investigation of the significance of peroxiporin expression as a factor in P-AscH^-^ therapy is warranted.

## Supporting Information

S1 Data Data and Analysis of the Signal Intensities Evaluated from Immunocytochemistry Staining for Peroxisomes. The excel file contains a worksheet titled "AQP 1, 3 & 8"; where, the raw data and subsequent analysis used to develop Fig 1 are presented. The file contains the evaluated signal intensities from immunocytochemistry images for AQP 1, 3 and 8, representative of the expression levels on H6c7 and MIA PaCa-2 cells. Target fluorescence intensity (from 10 images) per cell area for H6c7 and MIA PaCa-2 cells are included. Additionally, the statistical significance between protein expression (AQP 1, 3 or 8) and each cell type, determined through ANOVA (Single Factor), is also displayed on the worksheet along with the plot.
(XLSX)

S2 Data Raw Data for Flow Cytometry. The zip file contains the raw data used to generate Fig 2 which includes the detection for AQP3 on unmodified and siAQP3 MIA PaCa-2 cells.
(ZIP)

S3 Data Raw Data and Analysis for Rate of H_2_O_2_ Uptake Studies. The zip file contains all the data sets used to generate Fig 3 which represents the rate of H_2_O_2_ uptake per cell. Each excel file is named to clearly indicate the cell type/modification and case number. Each excel contains a read me tab, a tab of raw data and an additional tab containing the regression analysis.
(ZIP)

S4 Data Raw Data and Analysis for Clonogenic Assays. The excel file contains a worksheet titled "Normalization of Colony Counts" which contains the raw data and normalization for the colonies counted from the clonogenic studies of unmodified MIA PaCa-2, siAQP3 MIA PaCa-2, and H6c7 cells. The file also contains a worksheet titled "Analysis at each Dose" which provides the statistical significance of each cell comparison at each dose, determined through ANOVA (Single Factor), and is displayed to the right of the data sets.
(XLSX)
